# Health and political economy: building a new common sense in the United States

**DOI:** 10.1093/haschl/qxae041

**Published:** 2024-05-06

**Authors:** Victor Roy, Darrick Hamilton, Dave A Chokshi

**Affiliations:** National Clinician Scholars Program, Yale School of Medicine, New Haven, CT 06510-8088, United States; The Department of Economics, The Institute on Race, Power and Political Economy, The Public and Urban Policy Program, The New School, New York, NY 10011, United States; Colin Powell School for Civic and Global Leadership, The City College of New York, New York, NY 10031, United States; Bellevue Hospital, New York, NY 10016, United States

**Keywords:** political economy, capital, care, culture, health equity, determinants of health

## Abstract

The prevailing economic paradigm, characterized by free market thinking and individualistic cultural narratives, has deeply influenced contemporary society in recent decades, including health in the United States. This paradigm, far from being natural, is iteratively intertwined with politics, social group stratification, and norms, together shaping what is known as political economy. The consequences are starkly evident in health, with millions of lives prematurely lost annually in the United States. Drawing on economic re-thinking happening in fields like climate and law, we argue for a new “common sense” towards a health-focused political economy. Central to this proposed shift is action in 3 interconnected areas: capital, care, and culture. Re-orienting capital to prioritize longer-term investments, such as in public options for health care and baby bonds, can promote health and affirmatively include historically marginalized groups. Recognizing that caregiving is economically valuable and necessary for health, approaches like local cadres of community health workers across the United States would be part of building robust caregiving infrastructures. Advancing momentum in these directions, in turn, will require displacing dominant cultural narratives. As the health arena pursues change in the face of real obstacles, recent efforts reinvigorating industrial policy and addressing concentrated market power can serve as inspiration.

## Introduction

The prevailing economic paradigm has, for several decades, given primacy to free market thinking, underpinned by cultural narratives of individualism and deservingness that infuse our political discourse. This paradigm, often described as neoliberalism, has become a kind of “common sense,” shaping the contours of our society, akin to how the laws of gravity shape the curvature of space-time.^1^ Yet, our economy is anything but natural. Rather, the economy is inherently intertwined with politics, social group stratification (by identities such as race, gender, and national origin), and the norms and interests held by those that ostensibly benefit from the existing order. The iterative relationship among these, known as political economy, has profound implications for the well-being of individuals and society.^2^

The health implications of our existing political economy are stark, illustrated by the millions of “birthdays lost” each year in the United States, as well as persistent inequities in life expectancy, sometimes a decade or more, for Native, Black, and low-income Americans.^3,4^ These health crises demand a search for fresh responses that meet the scale of the challenges the country faces. We argue that adopting a “health and political economy” lens would enable the broader health field to build a new common sense, one that reorients economic strategy to focus on the health of people and communities.

## The need for a health and political economy lens

Such a common sense would build on, but also mark important departures from, a growing movement to recognize and address the “social determinants of health,” such as housing insecurity and everyday racism. While offering important conceptual orientation for tackling “upstream” drivers of health, social determinants interventions have, in practice, often experienced “downstream drift” by prioritizing harm mitigation in the face of unjust political and economic forces that otherwise passively unfold.^5^ For example, one predominant outgrowth of this movement has been the rise of multisector partnerships between health systems and other service providers to support patients experiencing the adverse impacts of food deserts or the housing crisis.^6,7^ While these partnerships are valuable in many instances, a health and political economy approach would instead direct attention not only to policies and strategies that mitigate harms caused by unjust arrangements but also to a more proactive focus on the political-economic forces that shape health ex ante.

This shift would be analogous to policy responses to wealth inequality, in which scholars have urged attention on upstream mechanisms for inclusive wealth building, like “baby bonds,” in addition to policies that focus on wealth redistribution.^8^ Baby bonds are publicly invested child trust accounts established at birth with the intent of redressing intergenerational poverty and the racial wealth gap. The accounts are rooted in the idea that financial capital is a critical ingredient for wealth building, and the accounts are progressively seeded such that children from lower resourced households receive the largest endowments. Once the child reaches adulthood, the resources from the publicly managed accounts could be used for wealth-building activities like higher education, homeownership, or entrepreneurship. This policy, a version of which is already being implemented in Connecticut, works to close the nation's racial wealth gap rooted in an unjust history that has not offered its Black citizens full economic participation.^9^

While proposing increased attention on analogous upstream health determinants is not new, action at the intersection of health and political economy has yet to become central to health research, policymaking, and practice.^10-12^ In arenas like climate and law, however, scholars, decision makers, and organizers have recently begun to embrace economic re-thinking and forge novel coalitions for action on challenges like renewable energy and monopoly power.^13^ Joining with these parallel efforts in other sectors, we view the existing relationship between the economy and health as not inevitable, but mutable in the hands of social agents.

## Capital, care, and culture: charting a direction for health and political economy

Three initial areas of focus could guide action on health and political economy: capital, care, and culture. “Capital” refers to the scale and quality of finance needed for health. Generating this finance will require taking on orthodoxies about markets and government that thrive in health policy, in which public finance is often oriented around socializing risk for private actors.^14^ For example, publicly financed programs like Medicare increasingly subsidize rewards for corporate shareholders, as observed in Medicare Advantage, while states seek to impose work requirements for Medicaid that limit patient access.^15^ Instead of exclusionary or short-term, extractive finance, a new consensus in this area would prioritize longer-term investment—for instance, in early childhood interventions—that also affirmatively includes historically marginalized groups.

A policy agenda regarding capital could, for example, protect and expand public provision of critical resources needed for health—so-called “public options” that co-exist with private ones. In the face of concentrated market power across health care, from the pharmaceutical industry to insurance companies, public options can lower prices and create more equitable access.^16^ A recent illustration is California's investments in a public option for drug manufacturing, by which the state is aiming to produce low-priced insulin.^17^ In addition to growing investment in other public options such as public hospitals, potential actions include setting stronger conditions on the substantial public financing that private entities like pharmaceutical and insurance companies receive.^18^ Such efforts would revive public capacity to shape markets towards population health goals, like eliminating hepatitis C through wider access to direct-acting antivirals. Reciprocally, wiser capital investment toward public health has both intrinsic virtues as well as broader economic benefits in a way that is consistent with sound industrial policy.

Alongside this focus on capital, a health and political economy strategy around “care” would cultivate systems and policies that invest directly in people's capabilities to realize health across the life course, including robust caregiving infrastructures. Reversal of the expanded child tax credit and crises of child and elder care are examples of the impact of undervaluing the resources needed for health at critical life stages. A new common sense would recognize and reward caregiving as an economically valuable activity.

Sturdier systems of home- and community-based care that center strong labor protections for the workers delivering this care are essential to support patients and families. Proposals for cadres of community health workers across the United States are one example in this vein.^19^ Mobilizing public finance for such workers would not only improve health but double as an engine for economic opportunity by creating jobs in communities that have been disproportionately impacted by mass incarceration, deindustrialization, and historic disinvestment. A broader care agenda would emphasize the economic resources needed for social support and opportunity in different life stages, such as paid family leave, child poverty reduction, baby bonds for younger Americans, and cash assistance for elder care.^20^

To inspire momentum in these directions, “culture” refers to the importance of changing hearts and minds about the mutual links between health and the economy. This area of focus appreciates the power of stories to shift attitudes, and the corresponding need to develop “displacing narratives” that supplant prevailing economic thinking. Discussions regarding universal health care serve as a case study. Narratives of “deservingness,” for example, can racialize and stigmatize public programs in ways that harm the health of low-income Americans across racial and ethnic groups, as observed with struggles over Medicaid expansion.^21^ One of the cultural and political tasks for advancing universal health care is to re-configure public investment to be intentionally inclusive, given the historical experience in which “universal” policies can otherwise leave behind stigmatized groups. This focus would also help account for persistent and widening racial disparities in health for Black people in the United States when attaining higher levels of socioeconomic status—a “paradox” linked to the physical and psychological costs of attempting to overcome highly stigmatized environments.^22,23^ Building power for many of the ideas outlined above, from baby bonds to universal health care, will require fresh stories paired with organizing that expands and consolidates popular support.

## The road ahead

The powerful narratives and vested interests that dominate our current political economy make change far from inevitable. Some observers will also reasonably point to polarization and partisanship as durable obstacles to advancing economic policy for health in the United States. This concern is particularly relevant considering the public finance required for some of the policies we have highlighted. To be sure, selecting strategic areas for public action in this political environment will be crucial for success, and decision-makers should contend with limits of government capacity in a given domain. Yet, decision-makers can also eschew traditional framings of spending on health as a cost and consider instead the substantial return on investment that policies like community health workers and baby bonds produce.^24-26^ Additionally, governments can mobilize substantial investment by addressing the regressive nature of our tax code, with evidence of extended longevity and reduced health inequities.^27,28^ Re-directing expensive and potentially wasteful government subsidies to corporate actors could yield another critical financing source.^29^

Seeking change in the face of these political and financing challenges, the health arena can derive inspiration from other fields. Responding to the decline in American manufacturing competitiveness, policymakers are reviving industrial strategy to proactively shape markets towards economic and social goals like clean energy.^30^ And with an economy composed of concentrated market power ranging from “big tech” to big agriculture, many in the legal arena are pursuing anti-monopoly strategies aimed at producing more innovation, lower prices, and worker protections.^31^ While these efforts face their own hurdles, they have brought fresh economic thinking and practice into the mainstream by breaking down walls between academia, policymakers, civil society, and culture-makers.^32^

The health arena needs a similarly ambitious approach. Confronted with stagnating life expectancy in the wake of an unprecedented pandemic, the country faces a stark choice. We can choose to treat prevailing economic thinking on health as a law like gravity, pulling us inexorably on our current path. Or we can build a new common sense, following the pioneering examples set by prior generations of Americans striving to improve the health of the nation, from mobilizing the sanitary movements of the 19th century to launching Medicare and Medicaid in the 20th. Our era calls for bold action on no less a scale.

## Supplementary Material

qxae041_Supplementary_Data
